# New Operation in Dental Science

**Published:** 1869-10

**Authors:** Q. L. Adams


### ARTICLE IX.

### *New Operation in Dental Science.*

Reported by Q. L. Adams, D. D. S.

An operation was performed in this city during the months of May and June, 1860, by Dr. C. E. Blake assisted by me, which is new in dentistry, and a description of which will be of interest to those in pursuit of dental science.

The gentleman upon whom the operation was performed, had been wearing a superior denture of artificial teeth, and having worn the remaining inferior teeth very much away, nearly to the margin of the gums, the four first inferior molars and second right bicuspid having been removed several years previously, the remaining portions of the dens sapientiæ had been forced very much forward.

May 13th. Applied the spray of sul. ether to the left dens sapientæ, and, when sufficiently benumbed, cut into the nerve cavity, which was but a slight distance, and extirpated the nerve with small barbed broaches designed for the operation, the sensation of pain being very slight. Owing to business engagements, the case remained under attention.

May 20th. After preparing and cutting threads with a screw top, inserted two screws of pure gold three-eighths of an inch in length, and one-eighth of an inch in diameter. As the posterior root extended back, the back screw had to be fitted in first, and curved, to bring the upper ends of the two parallel, where the threads of the screws had been re moved, and the two adjusted, filling up the threads and remaining space with Roberts' Os-artificial. The amount required was very little; as the screws nearly filled the orifice.

After the operation came a plate of pure gold, in thickness about twenty-nine by guage, and one sixteenth of an inch larger than the grinding surface of the tooth. Two openings were made in close proximity. The grinding surface of the tooth had worn down a little concave and uneven ; the gold plate was therefore put on and tapped down with instruments and mallet, to fit the surface perfectly by annealing it up, and a hard plate for service, composed of platina and gold, one-eighth of an inch in thickness and nearly the size of the tooth, fitted to the first plate, with the opening deeply counter-sunk around the ends of the screws. They were then taken off, and the two plates soldered together. There was then placed on the under surface of the thin plate, and of the same size, sixteen layers of gold foil, so as to make the adaptation impervious to the fluids of the mouth. The sharp corners of the tooth were then slightly taken off, in order to make a better fit, and to avoid any small fracture of the corners in the adaptation.

Everything now being ready and the usual precaution made to keep it dry, the compound plate or cap was put on in its place, and the upper ends of the gold screws were riveted down with the serrated pointed pluggers, and by the use of the mallet; and the remaining part of the counter-sunk cavity was filled with gold foil and sponge gold all solid and tight. The extended margin of the pure gold plate, together with the foil underneath, were then tamped down around the corner of the tooth. The perfect manner in which the plate of pure gold was tapped over and around the margin of the tooth, leaves no doubt of its security. Abont eight hours were consumed in the last operation.

May 24th. The corresponding molar on the right side was taken in hand, and the nerve-pulp extirpated,

May 28th. A succeesful operation was performed similar to the first. Subsequently, three bicuspids, and one canine were treated and capped in the same manner, with the exception that but one screw was inserted in each fang—some of which, gold foil was plugged around the screws in the fang to secure a perfect fastening.

On completion of these operations as above described, there was not any uneasines or pain experienced by the patient, except in the first bicuspid, on the right side, which had been treated for alveolar abscess eight years previously and was quite sensitive and painful during the operation, but yielded readily by the application of an astringent wash, and in a few days was restored to its former tone of health.

The crowns of several of these teeth, some eight months previously, had been built up solid by the use of the mallet, with adhesive gold, but after a few months' use it was discovered that they were rapidly wearing away, caused by the grinding force and hard surface of the artificial teeth coming in contact with the pure gold. This suggested the operation of capping with hard metal as the most permanent manner of prolonging their use.

The above operations being new in the practice of dentistry, and having taken an interest in their performance, I take the liberty to give them the name of Compound Cap Restoration.—*Pacific Med. & Surg. Journal.*

